# Attitudes and Defaults Save Lives and Protect the Environment Jointly and Compensatorily: Understanding the Behavioral Efficacy of Nudges and Other Structural Interventions

**DOI:** 10.3390/bs4030202

**Published:** 2014-07-17

**Authors:** Florian G. Kaiser, Oliver Arnold, Siegmar Otto

**Affiliations:** Department of Personality and Social Psychology, Otto-von-Guericke University, P.O. Box 4120, Magdeburg D-39016, Germany; E-Mails: oliver.arnold@ovgu.de (O.A.); siegmar.otto@ovgu.de (S.O.)

**Keywords:** environmental attitudes, conservation (ecological behavior), nudges, behavioral change, structural interventions, Campbell paradigm

## Abstract

A better understanding of when and why nudges (e.g., defaults, visibility or accessibility alterations) and other structural behavior-change measures work or fail can help avoid subsequent surprises such as unexpected political opposition. In this paper, we challenge the unilateral focus on structural interventions—which seemingly control people's behavioral decisions—as such a focus ignores the flipside—namely, attitudes or, as they are called in economics, preferences. We argue for a conceptual understanding of individual behavior that views personal attitudes and behavioral costs as its two separate compensatorily effective determinants. This classical understanding was reintroduced into attitude research as the Campbell paradigm. In the logic of the Campbell paradigm, a person's attitude becomes obvious in the face of the behavioral costs the person surmounts. Technically, individual attitudes reveal themselves in a set of cost-dependent transitively ordered performances. Behavioral costs in turn reflect the structural boundary conditions that are relevant as obstructive and/or supportive environmental forces that generically affect a specific behavior. So far, our research on people’s attitudes toward environmental protection has demonstrated that the Campbell paradigm—and thus its conceptual account of individual behavior—holds true for approximately 95% of the people in a given society.

## 1. Introduction

What drives the decision to donate one’s organs, install energy-efficient lightbulbs, or select a vegetarian dish? Johnson and Goldstein [1] would probably argue that structural cues, such as financial incentives or nudges (*i.e*., nonfinancial and noncompulsory structural forces such as defaults, visibility or accessibility alterations; see, e.g., [2]), often trigger these behavioral decisions or even produce these behaviors (see also [3,4]). Interestingly, however, nudges seem to work only when people do not hold pronounced attitudes ([1]; remember that attitudes are called preferences in economics, see [5]). In Johnson and Goldstein’s original words, “If preferences … are strong, we would expect defaults to have little or no effect” ([1], p. 1339). In other words, structural forces are expected to work if attitudes toward the issue of interest (e.g., protecting the environment, donating organs, connecting with nature) are absent or weak.

In our paper, we argue that the apparently *conditional* behavioral relevance of structural interventions (nudges, for example) is a misconception. We will demonstrate that the driving forces behind people’s behavioral decisions are attitudes (e.g., a person’s environmental attitude or attitude toward organ donation). By contrast, structural factors do not fuel behavioral decisions. Rather, they aggravate or alleviate individual performance as “behavioral costs” so to speak. Therefore, a unilateral focus on structural interventions—ignoring differences in the strength of individual attitudes—obstructs the understanding of when and why, for example, nudges work or fail.

To understand the efficacy and limits of nudges specifically and of structural forces in general, we will elaborate on a classical understanding of attitudes (e.g., [6]) that was recently reintroduced into attitude research as the Campbell paradigm (see [7]). The Campbell paradigm describes behavior as a function of two *compensatorily* effective factors: a person’s attitude and the structural boundary conditions that obstruct and/or support specific behavior (*i.e.,* as behavioral costs). With our paper, we wish to demonstrate the significance of this new paradigm to the understanding of behavioral change beyond its application as a model for environment‑relevant attitudes (e.g., [8]).

In the following section, we turn to Johnson and Goldstein’s [1] seminal *Science* article, which presents an illustrative example about the prevalence of organ donation in societies with distinct nonaction defaults. Whereas nonaction in societies with an opt-in default denotes a *refusal* to donate, nonaction in societies with an opt-out default denotes an *acceptance* of organ donation. We argue that both these implicit connotations of people’s inaction are inaccurate as they fail to reflect people’s attitudes toward organ donation. We believe that such misunderstandings of people’s attitudes carry the risk of subsequent surprises in the form of failed communal acceptance or even public resistance.

## 2. Apparent Nudging Effects

**Figure 1:**
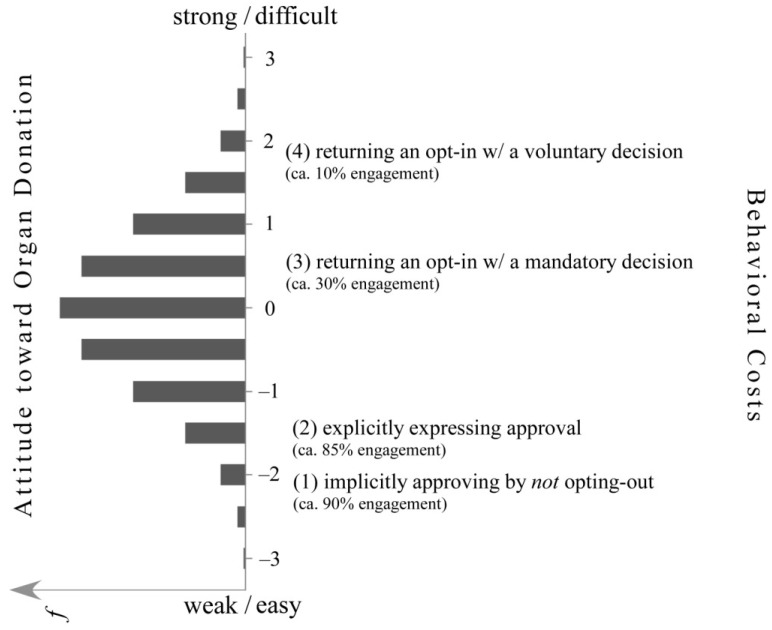
Offsetting specific behavioral costs with the strength of a person’s individual attitude toward organ donation*.* The horizontal bars display a prototypical distribution of the strength of people’s attitudes toward organ donation on a scale ranging from weak to strong. Correspondingly, organ-donation-relevant behaviors are ordered according to their cost-based difficulty on a scale ranging from easy to difficult.

Johnson and Goldstein [1] found that the probability (*p*_ki_) of person k becoming an organ donor (*i.e*., behavior i) is about 10% in societies with an opt-in default and about 90% in societies with an opt-out default. Evidently, if people are nudged into one of two defaults—remaining a nondonor (with the option to opt-in) or remaining a donor (with the option to opt-out)—the probability of becoming a donor changes dramatically (see Figure 1). By replacing an opt-in default with an opt-out default, societies can increase the proportion of organ donors by about 80%. How can implementing an opt-out rather than an opt-in procedure—involving two behaviors that carry apparently negligible and nearly identical behavioral costs—advance people’s likelihood of donating their organs so dramatically?

Interestingly, by replacing the voluntary opt-out choice with a mandatory active choice—making everybody send a return-card—the likelihood of donorship can be expected to decrease from 90% to approximately 30% (see Figure 1; based on [1], Footnote 28). Increasing the behavioral costs, obviously, leads to a loss of 60% of potential donors relative to the voluntary opt-out condition in which one is not required to answer. This loss seems indeed to speak of—as Johnson and Goldstein presume—the behavioral efficacy of nudges. Conceptually, as we argue, nudges are merely structural conditions that aggravate or alleviate, and they are not the driving forces behind people’s behavioral decisions, as could be concluded from Johnson and Goldstein’s [1] title (*i.e*., “Do Defaults Save Lives?”). Next, we describe a framework that explains individual performance (including behavioral decisions) as a compensatory relation of behaviorally effective attitudes (in our case, the attitude toward organ donation) and costs (involved with, e.g., either the opt-in or the opt-out default).

## 3. Identifying Attitudes

According to Campbell [9], a person’s esteem for an attitudinal object (e.g., organ donation) or the behavioral goal implied by the attitude (e.g., donating one's organs) becomes obvious in the face of behavioral costs, the environmental forces that obstruct or support a given behavior (see e.g., [10]). In other words, when people are trying to overcome such structural obstacles, expend a lot of effort, or tolerate a lot of distress when implementing a behavior (*i.e*., consenting to organ donation), we can presume that the person is highly committed to the behavioral goal implied by the attitude (*i.e*., organ donation). That is, we can say that the person's specific attitude toward organ donation is pronounced or his/her attitude toward organ donation is extremely favorable. Thus, it seems reasonable to assume that if a person is genuinely dedicated to organ donation, he/she would presumably (1) implicitly approve of organ donation by not returning an opt-out return-card, (2) explicitly express approval for organ donation (e.g., on a questionnaire; based on [1], p. 1338), (3) explicitly grant permission for organ removal by opting-in when the decision is mandatory, and (4) return a voluntary opt-in return-card (see Figure 1).

Conversely, when the experience of unpleasant sentiments is enough to stop a person from even expressing approval for organ donation on a survey, his/her attitude toward organ donation is expectedly fairly weak. According to this logic, a person’s attitude toward organ donation will manifest itself in the various behavioral options necessary to attain the goal implied by the attitude (*i.e.*, donating one’s organs). Thus, the stronger a person's attitude is, he/she will implement more behaviors and more demanding behaviors. Recognizing an attitude therefore requires the empirical identification of a set of behaviors that represent what people commonly do to implement the strength of a specific personal attitude (e.g., toward organ donation; see Figure 1). Note that empirically identifying sets of behaviors that are—due to their costs—transitively ordered is not a trivial matter as it can fail (for typical successful examples, see [11,12,13]).

## 4. Attitudes and Performance

In this view, an individual's attitude represents his or her particular esteem for organ donation (see Figure 1), and it is equated with the personal engagement likelihoods of the behaviors representing the very attitude [6]. In other words, the engagement likelihoods of the four specific behaviors depict the typical prevailing attitude toward organ donation in a given society. Hence, these likelihoods indicate that the mean organ donation attitude of people is pronounced enough that about 90% of the population will take no action—not even an action as small as sending back an opt-out card—to avoid being identified as an organ donor, provided that everybody else is assigned as well. However, if this assignment requires active engagement—when the opt-in alternative is applied—the situation changes dramatically. This becomes apparent in the drop to about 10% of the population who agree to be donors. Obviously, people's attitude toward organ donation is, in this example, on average not strong enough to even overcome the apparently marginal costs that accompany an opt-in default.

Thus, the distinct relative frequencies in Figure 1 imply three things: First, choosing to opt-in is not the same behavior as choosing not to opt-out because the former seems to involve more costs than the latter. Second, people who voluntarily opt-in hold much stronger attitudes toward organ donation than people who simply do not actively opt-out. Third, with only these two behaviors in sight, individual attitudes toward organ donation can hardly be assessed accurately.

### 4.1. Recognizing Effective Costs

The choices to either opt-in or not opt-out apparently do not carry the same actual costs and, thus, do not reflect identical structural forces—although the two behaviors appear quite similar. In their attempt to explain the observed differences in organ donation rates in societies with opt-in as opposed to opt-out defaults, Johnson and Goldstein [1] also came up with three cost-based explanations: effort, loss aversion or reference dependence, and implied endorsement.

Effort refers to the mental pain that may be involved when a person makes up his or her mind or to the physical labor (*i.e.*, seeking a mailbox) that is required when a person mails a response-card using the postal service. In other words, in their first explanation, Johnson and Goldstein focused on the personal aspects that correspond with structural costs (*i.e.*, environmental demands). These consist of the presumed personal expenditures that are involved when a person switches away from the default, that is, mental and physical effort.

Loss aversion, the second explanation, also refers to behavioral costs. In this explanation, a default and its presumed costs represent the cognitive reference. Any alternative (*i.e*., the nondefault) has to be profitable by comparison. In other words, the departure from the benchmark has to promise some benefits or substantively fewer costs than the default; otherwise, people’s aversion to loss—through not gaining anything—results in the persistence of the default [14].

The implied endorsement, the third explanation, refers to a specific set of behavioral costs. In this explanation, a default represents the normative reference with regard to the expected course of action (e.g., [15]). Any alternative (the nondefault) implies a departure from the majority position and, thus, the risk of social stigma. According to this perspective, we would expect a person’s attitude toward social conformity—provided such an attitude exists—to fuel the significance of defaults.

If behavioral costs are so crucial, empirically uncovering behavioral costs and detecting potentially behavior-effective boundary conditions becomes indispensable. In line with Johnson and Goldstein [1], we also suggest systematically comparing engagement likelihoods across different cities, regions, or societies as the measure of choice (see e.g., [10,16]). For example, turning off one's car at red traffic lights appeared to be relatively demanding—with a 2% likelihood—in Eindhoven, The Netherlands, compared with two German cities (with a 12–15% likelihood) and Zürich, Switzerland (with a likelihood of 41%; see [17]). From these numbers and from studying the locations, we learned that one effective nudge in The Netherlands might be to introduce a yellow warning light before traffic lights turn green, which is standard in Germany and Switzerland but was not in The Netherlands at the time of our study. An effective nudge to make people switch off their engines at red lights in Germany might, by contrast, consist of prompts to remind drivers to switch off their engines as found in Switzerland and originally introduced in a massive public campaign.

### 4.2. Insurmountable Attitudes

Sometimes nudges will not work. Children, for example, could not be nudged into replacing French fries with apples by making fries less and apples more accessible (see [18]). Increasing the behavioral costs of French fries by making them hard to reach cannot surmount the preexisting food preferences of most children. Likewise, OECD employees opposed decreased temperature defaults of 18 or 17 °C but tolerated defaults of 19 °C in their offices. Apparently, a reduction from 20 to 19 °C does not impinge on the thermal comfort preferences of most employees enough to draw counteraction, but a reduction to 18 or 17 °C, by contrast, makes a majority of employees rebel against the change [19].

As we presume, the behavior-effective driving forces behind people's behavioral decisions are individual attitudes (e.g., food-, thermal comfort-, or environmental protection preferences). In other words, structural influences, such as nudges, depend on the driving force of supportive attitudes (see Figure 1) even when the structural influences appear to control behavioral decisions or seem to yield behaviors as incentives or disincentives (see [2]). In other words, structural measures can aggravate or alleviate behavior as its costs, but they cannot instigate individual performance.

### 4.3. Offsetting Behavioral Costs with Attitudes

From knowledge of the behavioral costs people endure, we can stochastically (*i.e*., for large groups of people, such as societies) derive the specifically required strength of the attitude. Thus, we have to presume that persons who voluntarily opt-in (*i.e*., the comparatively most costly behavior #4 in Figure 1) hold much stronger attitudes toward organ donation than persons who exclusively do not actively opt-out (*i.e.*, the comparatively cost-free behavior #1). By raising the behavioral costs substantially relative to the not-opt-out condition (#1), about 80% of potential donors would be lost—the ones with a weaker attitude strength than the strength required by the specific behavioral costs involved—with the voluntary opt-in condition (#4). With fewer behavioral costs relative to the voluntary opt-in condition given by a mandatory opt-in decision (#3), only about 60% would be lost. This prediction is based on the fact that the average attitude toward organ donation in most societies is not strong enough to make the majority explicitly donate their organs—on the basis of voluntary or mandatory decisions (see Figure 1). Thus, to eventually understand the efficacy of nudges, we need to understand the inherent connection between attitudes and costs, that is, the connection between the psychological factor and the structural factor.

According to Figure 1, neither behavioral costs (*i.e*., the structural factor) nor attitudes (*i.e*., the psychological factor) can be understood in an absolute sense. They are relative to each other; they are interdependent. Attitudes become manifest in the behavioral obstacles they help people overcome, and conversely, costs become manifest in the strength of the attitude they require to be offset. If organ donation was inherently more important for most, we would find less resistance than the resistance represented by the opt-in conditions, resulting in more donors than only 10%–30%. Thus, with increasing costs, progressively stronger attitudes are required for a behavior to be actualized. Mathematically, the relation between behavioral costs and attitudes can be depicted by the following formula (for more details, see [7]):

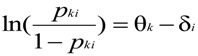
(1)


Formula 1 captures the natural logarithm of the ratio of the probability (*p*_ki_) that person k will engage in a specific behavior i relative to the probability that person k will not engage in behavior i (1-*p*_ki_) as a function of the arithmetic difference between the strength of k’s attitude (θ_k_) and the composite of the costs involved in realizing the specific behavior i (δ_i_). Note that here, people are distinguishable by the strength of their attitude (left side of Figure 1)—given the specific costs involved in realizing the attitude-relevant behaviors. Behaviors, by contrast, are distinguishable by how difficult they are to implement (right side of Figure 1)—given the attitude differences in a certain population. In other words, our conceptual understanding of attitudes fueling behavior in the presence of the obstructive and supportive forces depicted in Figure 1 represents a *compensatory* model of personal and structural forces. Thus, excessive impediments against a particular behavior can and must be compensated for by strong attitudes, whereas weak attitudes can still be overcome with even further reductions in behavioral costs. However and although the Campbell paradigm seems to provide a suitable conceptual framework for understanding the efficacy of structural interventions (e.g., in the form of nudges) and their failures, we have to be aware that the paradigm contradicts the dominant view of *conjunctively* effective personal and structural forces in social and much of environmental psychology.

Whereas the Campbell paradigm describes behavioral costs and, thus, structural interventions as generically effective (at least within socio-cultural contexts such as societies; see [7]), modern-day psychology expects attitudes and costs to interact in a statistical sense when conceptualizing the origins of behavior (e.g., [20]). In other words, attitudes and costs are thought to moderate each other's behavioral relevance (see e.g., [21]). The implication of this generally assumed interaction hypothesis is that people are expected to respond rather inconsistently (*i.e*., not uniformly) to structural interventions such as nudges or even economic incentives. Effectively controlling behavioral decisions with structural measures, however, requires their effectiveness to be more or less uniform. Thus, the Campbell paradigm would be a promising alternative to the currently dominant conjunctive model of personal and structural forces in social and much of environmental psychology.

Along these lines, Kaiser and Schultz [21] tested three different conjunctive models previously proposed in the literature. All three speak of a moderating influence of behavioral costs on the environmental attitude-ecological behavior relation: a positive linear model—where the strength of the attitude-behavior relation increases with behavioral costs; a negative monotonic model; and a curvilinear model with a marked drop in the strength of the attitude-behavior relation for easy and difficult behaviors. Contrary to all three models, behavioral engagement turned out to be an unmoderated, thus, unconditional function of environmental attitude (*r* = 0.54) in a pooled sample of *N* = 3338 participants (as long as the behaviors were not too easy (*i.e*., *p* > 0.95) or too difficult (*i.e*., *p* < 0.05) and, thus, their distributions remained within technically sensible boundaries for parametric statistics). Kaiser and Schultz’s findings simultaneously challenge the assumption of conditional effectiveness of either attitudes depending on structural forces or of structural forces depending on attitudes. By contrast, however, the conditional effectiveness of structural forces as depending on weak attitudes is commonplace in the literature on nudging (see, e.g., [1]).

Note that any systematic influence not accounted for by the model—such as moderators, alternative attitudes (e.g., [15]), or differentially effective behavioral costs—statistically reduces the fit of the model and, thus, eventually falsifies the model. So far, our research on environmental attitudes (*i.e*., the attitude toward environmental protection: see e.g., [8]) has demonstrated that the Campbell paradigm holds true for approximately 95% of the people in a given society in this behavioral domain at least. In other words, anticipating engagement in a specific behavior—on the basis of a compensatory relation between rather uniformly effective behavioral costs and individual attitudes—has turned out to be erroneous in only negligible proportions of individuals.

### 4.4. Three Examples of the Cost-Attitude Relation

If the Campbell paradigm provides a suitable framework for understanding individual behavior, we should be able to stochastically derive the specifically required strength of the attitude from the behavioral costs that people are willing to overcome. In our own research, we expectedly found that the cost of being a vegetarian—a highly effective, but in most Western societies (with a 5%–10% likelihood), quite a costly way to protect the environment—had to be offset by progressively more favorable attitudes toward protecting the environment. As it turned out, nonvegetarians held significantly weaker environmental-protection attitudes than vegetarians (see [7]).

**Figure 2:**
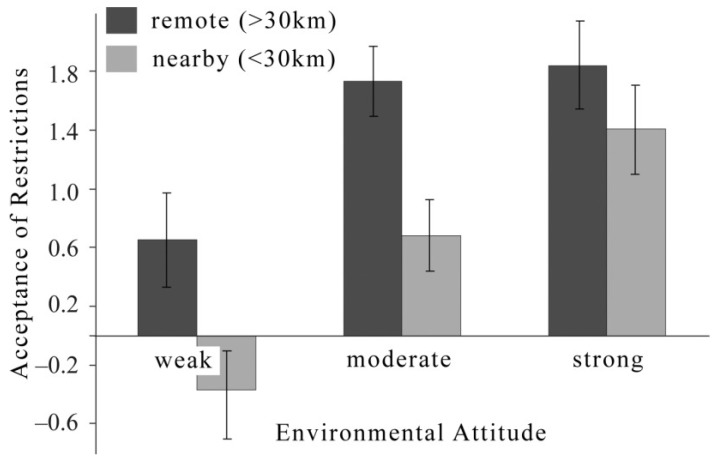
Remoteness-dependent acceptance of nature-preserve-related restrictions (NIMBY) moderated by people’s attitudes toward environmental protection (*i.e*., environmental attitude). Vertical bars indicate 95% confidence intervals.

In another study, we explored the NIMBY—not-in-my-backyard—phenomenon (see [22]). NIMBY indicates that people are better prepared to accept the restrictions and regulations (*i.e*., costs) that come with nature preserves the further they live from any preserve. When *not* at risk of being affected—by living more than 30 kilometers from any preserve—people generally accept the related restrictions (*i.e*., costs) unless they have comparatively weak attitudes toward environmental protection. When at risk of being affected—with increasing behavioral costs—restrictions are accepted with progressively increasing environmental-protection attitudes. Hence, people who are affected by restrictions need rather pronounced environmental-protection attitudes to accept preserve restrictions to an extent that is similar to those of people who are not at risk of being affected (see Figure 2).

**Figure 3:**
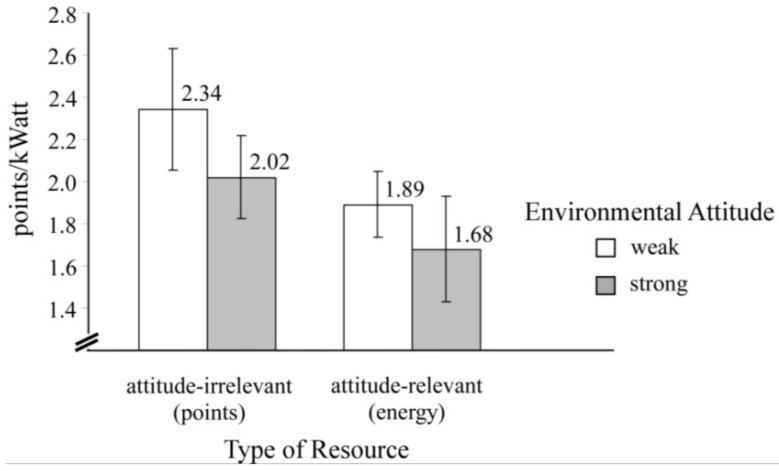
Points/kWatt claimed as a function of people’s environmental attitude and of the attitudinal relevance of the common resource. Vertical bars indicate 95% confidence intervals.

Such survey-based examples of the compensatory relation of a person’s attitude strength and the involved behavioral costs were corroborated in still another study that involved overt behavioral engagement in a laboratory experiment [23]. Claims for an attitude-irrelevant (*i.e*., points) compared with an attitude-relevant (*i.e*., energy) common resource turned out to be a function of a person’s environmental attitude. Specifically, Kaiser and Byrka [23] found higher claims for the attitude-irrelevant resource, irrespective of attitude strength (see Figure 3). Conversely, they also found that persons with strong—as compared with weak—environmental protection attitudes removed relatively less of the common resources.

## 5. Conclusions

In this paper, we challenged the unilateral focus on nudges and other behaviorally relevant structural forces—or in the language of the Campbell paradigm—behavioral costs. As we argue, ignoring the flipside of costs—namely attitudes—obstructs our understanding of why and when structural interventions generally work or fail. We advocate the conceptual understanding depicted in Figure 1 in which attitudes become manifest in the behavioral obstacles they help people overcome, and conversely, costs become manifest in the strength of the attitude they require to be offset. So far, our research on people's attitudes toward environmental protection has demonstrated that the Campbell paradigm holds true for approximately 95% of the people in a given society.

Effectively controlling behavioral decisions with structural measures requires their effectiveness to be more or less uniform. The Campbell paradigm describes behavioral costs and, thus, structural/environmental and personal forces as generically and jointly but compensatorily effective. As such, it provides a promising alternative to the currently dominant assumption of a person-environment interaction in social and much of environmental psychology.
